# Structurally constrained and pathology-aware convolutional transformer generative adversarial network for virtual histology staining of human coronary optical coherence tomography images

**DOI:** 10.1117/1.JBO.29.3.036004

**Published:** 2024-03-25

**Authors:** Xueshen Li, Hongshan Liu, Xiaoyu Song, Charles C. Marboe, Brigitta C. Brott, Silvio H. Litovsky, Yu Gan

**Affiliations:** a Stevens Institute of Technology, Department of Biomedical Engineering, Hoboken, New Jersey, United States; b Stevens Institute of Technology, Semcer Center for Healthcare Innovation, Hoboken, New Jersey, United States; c Icahn School of Medicine at Mount Sinai, New York, New York, United States; d Columbia University Medical Center, New York, New York, United States; e The University of Alabama at Birmingham, School of Medicine, Birmingham, Alabama, United States

**Keywords:** optical coherence tomography, histopathology, virtual histology, generative adversarial network, transformer

## Abstract

**Significance:**

There is a significant need for the generation of virtual histological information from coronary optical coherence tomography (OCT) images to better guide the treatment of coronary artery disease (CAD). However, existing methods either require a large pixel-wise paired training dataset or have limited capability to map pathological regions.

**Aim:**

The aim of this work is to generate virtual histological information from coronary OCT images, without a pixel-wise paired training dataset while capable of providing pathological patterns.

**Approach:**

We design a structurally constrained, pathology-aware, transformer generative adversarial network, namely structurally constrained pathology-aware convolutional transformer generative adversarial network (SCPAT-GAN), to generate virtual stained H&E histology from OCT images. We quantitatively evaluate the quality of virtual stained histology images by measuring the Fréchet inception distance (FID) and perceptual hash value (PHV). Moreover, we invite experienced pathologists to evaluate the virtual stained images. Furthermore, we visually inspect the virtual stained image generated by SCPAT-GAN. Also, we perform an ablation study to validate the design of the proposed SCPAT-GAN. Finally, we demonstrate 3D virtual stained histology images.

**Results:**

Compared to previous research, the proposed SCPAT-GAN achieves better FID and PHV scores. The visual inspection suggests that the virtual histology images generated by SCPAT-GAN resemble both normal and pathological features without artifacts. As confirmed by the pathologists, the virtual stained images have good quality compared to real histology images. The ablation study confirms the effectiveness of the combination of proposed pathological awareness and structural constraining modules.

**Conclusions:**

The proposed SCPAT-GAN is the first to demonstrate the feasibility of generating both normal and pathological patterns without pixel-wisely supervised training. We expect the SCPAT-GAN to assist in the clinical evaluation of treating the CAD by providing 2D and 3D histopathological visualizations.

## 1 Introduction

Coronary artery disease (CAD) is the narrowing of coronary arteries caused by a build-up of atherosclerotic plaques. As the most common type of heart disease, CAD leads to one in seven deaths in the United States.^1^ Optical coherence tomography (OCT) has been recognized as a valuable tool for imaging coronary tissue structures due to its high-resolution capabilities.^2^ However, real-time interpretation of OCT images requires a significant amount of expertise and prior training. Additionally, the power of OCT interpretation, especially of the pathological region, is hindered by the lack of histopathological correlation. At present, direct histopathological analysis requires an invasive and time-consuming evaluation that involves post-mortem tissue examination. The use of multiple reagents in histopathology can also lead to detrimental effects on tissue imaging. Histopathological analysis is not suitable for clinical use in patients, who require real-time tissue characterization of coronary arteries.

**Fig. 1:**
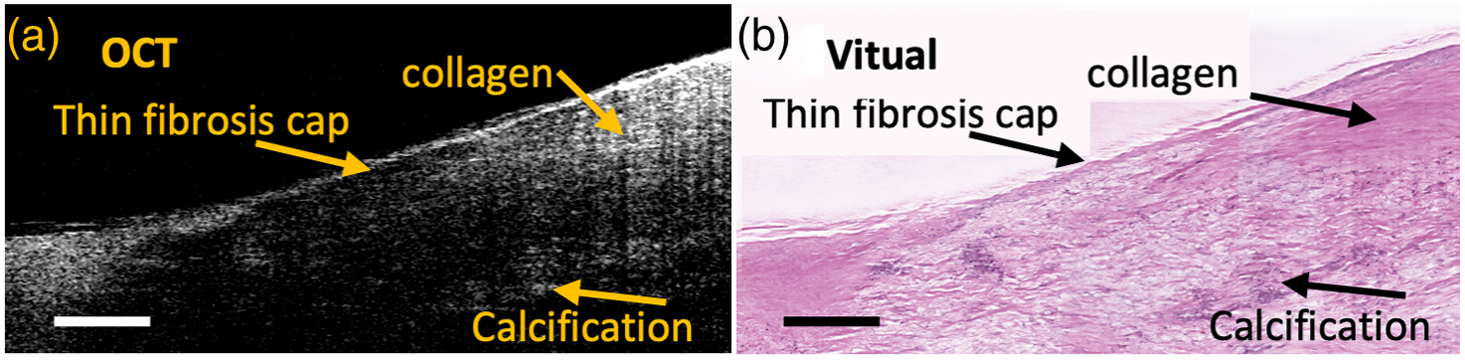
(a) Example OCT image of a human coronary sample. (b) Virtual stained histology image generated from the example OCT images in panel (a). The scale bar: 500  μm.

Incorporating histopathological visualization into real-time OCT imaging holds great potential to complement OCT with histopathological visualization. A typical example of generating virtual stained histology images from OCT images of human coronary arteries is shown in Fig. 1. To date, there are limited frameworks developed to generate virtual stained histology from OCT images.^3^^,^^4^ Winetraub et al. used Pix2Pix Generative Adversarial Networks (GANs) to generate virtual stained hematoxylin and eosin (H&E) histology for human skin tissues.^3^ However, Pix2Pix GAN for virtual staining requires a pixel-wisely paired OCT and H&E image dataset. The creation of a pixel-wisely paired dataset demands a significant investment of resources and labor, including the embedding of samples in fluorescent gel, photo-bleaching, and manual fine alignment.^3^ Such a method also lacks generalizability to blood vessels, which are deformable soft tissue. Our previous method^4^ demonstrates the capability to segment the three-layer structure (i.e., intima, media, and adventitia) in both OCT and H&E images, thereby generating virtual stained images optimized for different layers in human coronary. However, current performance has not been optimal if there are pathological patterns, such as calcium and lipid accumulation, that alter the typical three-layer structure of human coronary arteries.

To generate pathological-related regions from an unpaired dataset, we propose a structurally constrained pathology-aware convolutional transformer GAN (SCPAT-GAN) to generate virtually stained H&E histology images from OCT images. The proposed SCPAT-GAN incorporates two key components to enhance image quality for both normal and pathological coronary samples: a structural constraining module and a pathology awareness module. In summary, our main contributions include the following.

1. We propose a convolutional transformer-GAN structure for virtual H&E staining of human coronary arteries based on OCT. This generative method does not require pixel-wise mapping in the training dataset.
2. We incorporate structural constraining and pathology awareness modules for virtually staining coronary arteries with both normal three-layer structures and pathological patterns.
3. We conduct extensive quantitative experiments, including a blind test involving pathologist, to demonstrate that high-quality virtual histology images are generated.

## 2 Methods

### 2.1 Design of SCPAT-GAN

#### 2.1.1 Network architecture

**Fig. 2:**
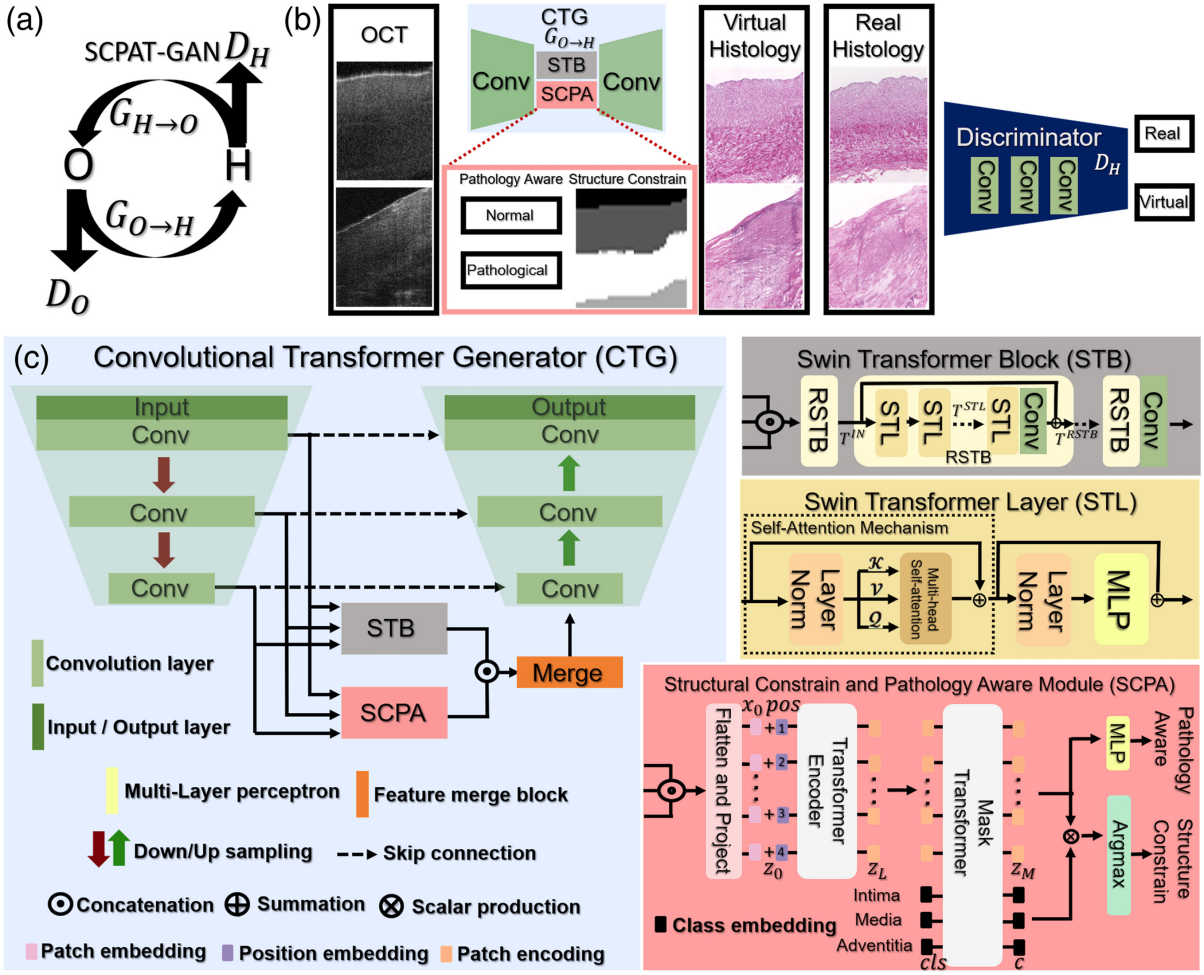
(a) The design of the SCPAT-GAN. (b) The scheme of $G_{O\to H}$ and $D_{H}$. The $G_{O\to H}$ performs virtual staining based on OCT images. The $D_{H}$ distinguish the virtual histology images from real histology images. The SCPA module guide the virtual staining process by performing structural constraining and pathology awareness functions. (c) Details of the convolutional transformer generator (CTG). The multi-scale features are fed to STB and SCPA modules for virtual staining.

The design of SCPAT-GAN is shown in Fig. 2. The SCPAT-GAN consists of two convolutional transformer generators ($G_{O\to H}$ and $G_{H\to O}$) and two discriminators ($D_{H}$ and $D_{O}$). The transformer structure possesses self-focus mechanisms that provide the global context of a given data sample even at the lowest layer. $G_{O\to H}$ transfers images from OCT domain to the histology domain; $G_{H\to O}$ transfers images from the histology domain to the OCT domain. The two generators share a similar structure. $D_{H}$ is the discriminator for histology images and $D_{O}$ is the discriminator for OCT images. Symbols O and H stand for OCT and histology images respectively.

The convolutional transformer generators ($G_{O\to H}$ and $G_{H\to O}$) take advantage of U-Net^5^ like structure to extract multi-scale features. The multi-scale features are sent to Swin transformer block (STB) and structural constraint and pathology aware (SCPA) module. The STB is a deep neural network architecture that employs multiple residual Swin transformer sub-blocks (RSTBs) to extract features from input data. The RSTBs contain various Swin transformer layers (STLs)^6^ that facilitate local attention and cross-window interaction learning. The feature extraction process of RSTBs is expressed as: $T^{\mathrm{RSTB}}=\mathrm{Conv}(F^{\mathrm{STL}}+T^{\mathrm{IN}})$, where $F^{\mathrm{STL}}$ denotes the features generated from STLs, Conv represents 2D convolutional layer with a kernel size of 3×3, and $T^{\mathrm{IN}}$ represents the input feature of RSTBs. Each STL comprises components including layer normalization, multi-head self-attention (MHA) modules, residual connections, and a two-level multilayer perceptron (MLP) with Gaussian error linear unit (GELU) non-linearity. Given an input of size H×W×C, the STL will reshape the input to the feature map of $\frac{\mathrm{HW}}{N}\times N\times C$ by partitioning the input into non-overlapping windows of N patches, where $\frac{\mathrm{HW}}{N}$ is the total number of the windows. For a local window feature X∈R, the query Q, key K, and value V matrices are computed as $Q=XP_{Q}$, $K=XP_{K}$, and $V=XP_{V}$, where Q, K, $V\in R^{N\times d}$. The $P_{Q}$, $P_{K}$, and $P_{V}$ are projection matrices shared across different windows. The self-attention of each head can be calculated as: $\text{Attention}(Q,K,V)=\text{SoftMax}(\frac{QK^{T}}{\sqrt{d}}+B)V$, where d denotes the query dimension; N stands for the number of patches in a window; and $B\in R^{\left(2N-1)\times (2N+1\right)}$.

#### 2.1.2 Structural constraining and pathology awareness

The SCPA module is based on a transformer encoder-decoder architecture, which guides the virtual staining procedure. The SCPA module performs structural constraining and pathology awareness functions by segmenting the human coronary layers and classifying the types of coronary samples (normal or pathological). The multi-scale features are split into a sequence of patches $x=[x_{1},\ldots ,x_{N}]\in R^{N\times P^{2}\times C}$, where (P,P) stands for the patch size, N represents for the number of patches, and C is the number of channels of the multi-scale features. The patches are flattened and then linearly projected to an embedding sequence $x_{0}=[E_{x_{1}},\ldots ,E_{x_{N}}]\in R^{N\times d}$, where d is the embedding dimension. Learnable position embeddings $\mathrm{pos}=[{\mathrm{pos}}_{1},\ldots ,{\mathrm{pos}}_{N}]\in R^{N\times d}$ are added to the sequence of patch embeddings to generate the tokens $z_{0}=x_{0}+\mathrm{pos}$ for Encoder. The Encoder maps the input sequence $z_{0}$ to $z_{L}=[z_{L_{1}},\ldots ,z_{L_{N}}]$, which is an encoding sequence containing contextualized information of multi-scale features.

The SCPA module is designed to be aware of pathology patterns as well as maintain and constrain the normal structure of coronary samples. In the case of normal coronary samples, the $z_{L}$ is decoded to a segmentation map $s\in R^{H\times W\times K}$, where K=3 and represents the three-layer structure of human coronary arteries. The segmentation map is acquired by the SCPA module, taking the scalar production between patch embeddings $z_{M}$ and class embeddings c: $\text{Segmentaion}=z_{M}c^{T}$, where $z_{M}$ is acquired by decoding $z_{L}$, and c is acquired by decoding a set of three randomly initialized learnable class embeddings [${\mathrm{cls}}_{\text{Intima}}$, ${\mathrm{cls}}_{\text{Media}}$, ${\mathrm{cls}}_{\text{Adventitia}}$] corresponding to the three coronary layers. In the case of diseased coronary samples, the patch embeddings $z_{M}$ are sent to a two-level MLP for classification between normal and pathological coronary images: $\text{Classification}=\mathrm{MLP}(z_{M})$. Also, the patch embeddings $z_{M}$ is concatenated to the extracted features from STB and then merged and up-sampled for OCT → Histology and Histology → OCT conversion.

#### 2.1.3 Loss function

The loss function L of SCPAT-GAN consists of five terms, which are adversarial loss $L_{\mathrm{adv}}$, cycle-consistency loss $L_{\text{cycle}}$, embedding loss $L_{\text{embedding}}$, structural constraint loss $L_{\mathrm{SC}}$, pathology awareness loss $L_{\mathrm{PA}}$

$$L(G_{O\to H},G_{H\to O},D_{H},D_{O},G_{O\to H}^{\mathrm{SC}},G_{H\to O}^{\mathrm{SC}},G_{O\to H}^{\mathrm{PA}},G_{H\to O}^{\mathrm{PA}})\;=L_{\mathrm{adv}}(G_{O\to H},D_{H})+L_{\mathrm{adv}}(G_{H\to O},D_{O})\;+\alpha L_{\text{cycle}}(G_{O\to H},G_{H\to O})+\beta L_{\text{embedding}}(G_{O\to H},G_{H\to O})\;+\gamma L_{\mathrm{SC}}(G_{O\to H}^{\mathrm{SC}},G_{H\to O}^{\mathrm{SC}})+\iota L_{\mathrm{PA}}(G_{O\to H}^{\mathrm{PA}},G_{H\to O}^{\mathrm{PA}}).$$ (1)

We follow the definition of $L_{\mathrm{adv}}$ and $L_{\text{cycle}}$ made by Zhu et al.^7^ and the definition of $L_{\text{embedding}}$ made by Liu et al.^8^ α, β, γ, and ι are hyper-parameters. $G_{O\to H}$ and $G_{H\to O}$ are two generators that generate virtual histology images from OCT images and virtual OCT images from histology images respectively. $G_{O\to H}^{\mathrm{SC}}$, $G_{H\to O}^{\mathrm{SC}}$, $G_{O\to H}^{\mathrm{PA}}$, and $G_{H\to O}^{\mathrm{PA}}$ are the SCPA modules for performing structural constraining and pathology awareness functions in the generators. The $L_{\mathrm{SC}}$ is implemented by segmentation loss

$$L_{\mathrm{SC}}=E_{H}[-S_{H}^{-1}\overset{s_{H}}{\underset{n=1}{\sum }}\overset{C}{\underset{c=1}{\sum }}y_{H}^{n,c}\;\log(G_{O\to H}^{\mathrm{SC}}(O))]+E_{O}[-S_{O}^{-1}\overset{s_{O}}{\underset{n=1}{\sum }}\overset{C}{\underset{c=1}{\sum }}y_{O}^{n,c}\;\log(G_{H\to O}^{\mathrm{SC}}(H))],$$ (2)

where $S_{H}$ and $S_{O}$ stand for the number of pixels in segmentation maps. $y_{H}^{n,c}$ and $y_{O}^{n,c}$ are the ground-truth pixel labels of different coronary layers for H&E and OCT images, respectively. C stands for the number of categories of the coronary layers (c=3). The $L_{\mathrm{PA}}$ is implemented by classification loss

$$L_{\mathrm{PA}}=E_{H}[-y_{H}^{p}\;\log(G_{O\to H}^{\mathrm{PA}}(O))+(1-y_{H}^{p})\log(1-G_{O\to H}^{\mathrm{PA}}(O))]\;+E_{O}[-y_{O}^{p}\;\log(G_{H\to O}^{\mathrm{PA}}(H))+(1-y_{O}^{p})\log(1-G_{H\to O}^{\mathrm{PA}}(H))],$$ (3)

where $y_{H}^{p}$ and $y_{O}^{p}$ are the ground-truth labels for pathology samples. We aim to solve the following minmax optimization problem

$$G_{O\to H}^{*},G_{H\to O}^{*}=\arg\;\min\;\max\;L(G_{O\to H},G_{H\to O},D_{H},D_{O},G_{O\to H}^{\mathrm{SC}},G_{H\to O}^{\mathrm{SC}},G_{O\to H}^{\mathrm{PA}},G_{H\to O}^{\mathrm{PA}}).$$ (4)

## 3 Experiments

### 3.1 Experimental Settings

#### 3.1.1 Experimental dataset

Human coronary samples were collected from the School of Medicine at the University of Alabama at Birmingham (UAB). Specimens were imaged via a commercial OCT system (Thorlabs Ganymede, Newton, New Jersey). A total of 194 OCT images were collected from 23 patients with an imaging depth of 2.56 mm.^9^ The pixel size was 2  μm×2  μm within a B-scan. The width of the images ranged from 2 mm to 4 mm depending on the size of sample. After OCT imaging, samples were processed for H&E histology at UAB.

We rescale the H&E images in the Aperio ImageScope software to enforce a pixel size of 2  μm×2  μm. Among the dataset, 112 OCT images are from normal samples with the three-layer structure (i.e., intima, media, and adventitia); 82 OCT and H&E images contain pathological patterns. At the pixel level, we pixel-wisely label the structure (e.g., the layer structure) in a subset of OCT and H&E images for training purposes. At the image level, we label each OCT or H&E image as the pathological OCT or normal. The OCT and H&E images are divided into non-overlap patches with a size of 368×368. We randomly flip the patches from left to right for data augmentation. The training set contains 4297 OCT image patches and 4297 H&E image patches.

#### 3.1.2 Implementation details

We adopt three convolution and transpose convolution layers with a stride of two for building a U-Net like structure for generating multi-scale feature maps. For the STB, we follow the design in our previous work.^6^ Our design of SCPA module is inspired by the Segmenter model.^10^ But different from the Segmenter,^10^ we design the SCPA module to be capable of performing both segmentation and classification tasks, which suits our need for structural constraining and pathology awareness functions during virtual staining. The SCPAT-GAN is implemented by Pytorch. For training, the hyperparameters α, β, γ, and ι are set to 1, 0.2, 5, and 5 empirically. The pixel values of OCT and H&E images are scaled to [0, 1]. The batch size is 9. The learning rate is initialized as $10^{-4}$, followed by a linearly decaying decay for every 2 epochs. In total, the SCPAT-GAN is trained 10,000 epochs to ensure convergence. The experiments are carried out on an RTX A6000 GPU.

#### 3.1.3 Metrics

We measure the similarity of pairs of virtual stained histology and real histology images using reference-free metrics including Fréchet inception distance (FID)^11^ and perceptual hash value (PHV).^8^ The FID is defined as

$$\mathrm{FID}=|\mu (G_{O\to H}(O))-\mu (H){|}^{2}-Tr(\sum G_{O\to H}(O)+\sum H-2\sqrt{\sum G_{O\to H}(O)\sum H}),$$ (5)

where $\mu (G_{O\to H}(O))$ and μ(H) are the magnitudes of the virtual stained and real histology images; Tr is the trace of the matrix; $\sum G_{O\to H}(O)$ and ∑H are the covariance matrix of the virtual stained and real histology images. The PHV is defined as

$$\mathrm{PHV}=\frac{1}{N}\sum H[|\mathrm{avg}(F_{i}(G_{O\to H}(O)))-\mathrm{avg}(F_{i}(H))|-T,$$ (6)

where N is the total number of extracted featuremap, $F_{i}$ represents the featuremap extracted from i’th layer of ResNet-101, avg is the average pooling operation that turns 3-D features into 1-D features, H is the unit step function, and T is a preset threshold. We use the three variations of PHV scores (i=1, PHV1), (i=2, PHV2), and (i=3, PHV3) which are extracted from different levels i of ResNet-101. We set T to be 0.02.

Also, we designed a protocol to involve two pathologists (Dr. Silvio H. Litovsky, referred to as pathologist A; and Dr. Charles C. Marboe, referred to as pathologist B) to evaluate the quality of the virtual stained H&E images. Real and virtual stained H&E images are given to the pathologists, who are blinded to the true labels, to make predictions. The two pathologists work independently from each other. We compare the prediction results from the pathologist with the true labels, following the setup of the visual Turing test.^12^^,^^13^

### 3.2 Results and Discussion

#### 3.2.1 Quantitative analysis

The quantitative results (calculated by three-fold cross-validation) of SCPAT-GAN, as well as two start-of-art methods, for generating virtual stained H&E are shown in Table 1. Compared to our previous method (Coronary-GAN^4^) and Cycle-GAN, the SCPAT-GAN generates virtual stained H&E images of better quality, with lower FID scores and higher PHV scores, for normal, pathological, and the whole dataset. Those scores indicate that virtual stained histology and real histology are perceptually similar. Moreover, we have two experienced pathologists with more than 30 years of experience to evaluate the quality of virtual stained H&E images. The pathologists, who are blind to the true labels, manually identify if an image is real or virtual.

**Table 1:** The FID and PHV scores of SCPAT-GAN, Coronary-GAN, and Cycle-GAN. The PHV scores calculated from different levels of the feature maps PHV1, PHV2, and PHV3. We report evaluation results for normal, pathological, and the whole dataset. All the results are calculated using three-fold cross-validation.

Metrics		Method		
		SCPAT-GAN	Coronary-GAN	Cycle-GAN
FID↓	Normal	**155.24**	205.02	253.15
	Pathological	**196.16**	275.47	315.92
	Whole	**175.70**	238.74	284.53
PHV1↑	Normal	**57.48**	55.49	50.87
	Pathological	**57.35**	40.87	40.09
	Whole	**57.41**	48.18	45.48
PHV2↑	Normal	**53.00**	51.39	52.19
	Pathological	**71.85**	58.69	56.44
	Whole	**62.42**	55.17	54.31
PHV3↑	Normal	**52.82**	51.41	48.67
	Pathological	**53.05**	47.17	48.68
	Whole	**52.93**	49.29	48.67

Note: The best performance results are highlighted in bold.

**Fig. 3:**
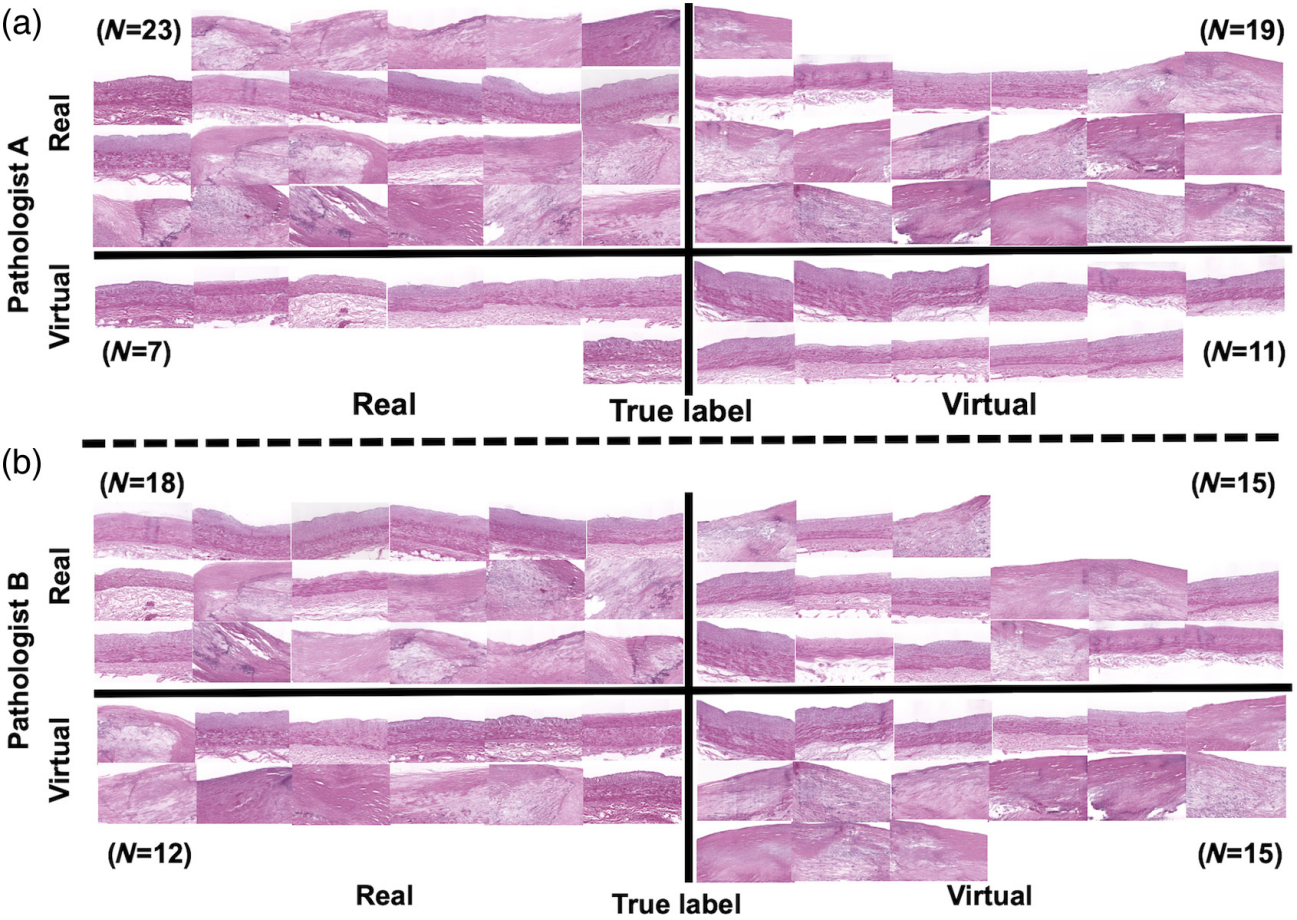
Result of pathologist’s evaluation of real and virtual stained H&E images. The number of images (N) in each quadrant is attached. (a) Evaluation results from pathologist A. (b) Evaluation results from pathologist B.

The results of pathologists’ evaluation are shown in Fig. 3. Among the total 60 images (half virtual and half real), over half of them (42 images by pathologist A and 33 images by pathologist B) are deemed as “real.” For the virtual stained images, more than half (19 images) are deemed as “real” by pathologist A and half (15 images) are deemed as “real” by pathologist B. We calculated the accuracy (pathologist A: 0.56; pathologist B: 0.55), precision (pathologist A: 0.54; pathologist B: 0.54) values of the evaluation results from the two pathologists. We compare the evaluation results with that of random guessing (in theory, accuracy and precision should be 0.5 for an observer who is making choices randomly). We found that the average accuracy (0.55) and precision values (0.55) are close to that of random guessing. The average sensitivity (0.68) is higher, which indicates that the pathologists are capable of identifying real histology images. However, the average specificity (0.43) is lower, which means the virtually stained images are less likely to be identified. Thus, the quality of virtually stained images is close to that of real histology images according to pathologists’ justification. Moreover, the intraclass correlation coefficient (ICC) between the evaluation results of the two pathologists is 0.014, which means a low interreader agreement because the images are indistinguishable.

#### 3.2.2 Ablation study

We perform an ablation study by removing the structural constraining (PAT-GAN) or pathology awareness functions (SCT-GAN) or both (T-GAN). The models are retrained and compared with the design of SCPAT-GAN. The results are reported in Table 2. When both structural constraining and pathological awareness models are equipped, SCPAT-GAN reaches the best performance. The ablated model without the structure constraining and pathological awareness modules (T-GAN) reports a compromised performance.

**Table 2:** The ablation study. We remove the pathological awareness module (SCT-GAN), structural constraining module (PAT-GAN), and both modules (T-GAN). The ablated models, SCT-GAN, PAT-GAN, and T-GAN, are retrained.

Metrics	FID↓	PHV1↑	PHV2↑	PHV3↑
SCPAT-GAN	**175.70**	**57.41**	**62.42**	**52.93**
SCT-GAN	177.60	53.59	59.28	51.33
PAT-GAN	176.96	53.57	59.94	50.45
T-GAN	203.24	47.00	54.32	47.48

Note: The best performance results are highlighted in bold.

#### 3.2.3 Qualitative analysis

**Fig. 4:**
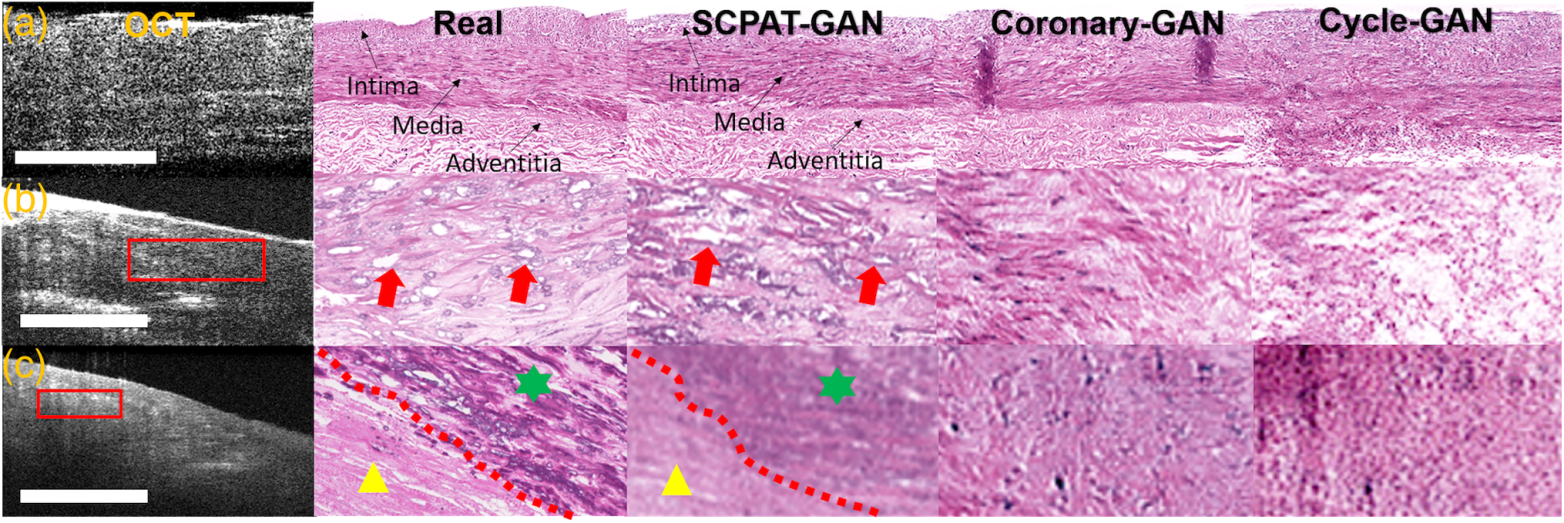
Visual inspection of virtual stained H&E images generated by SCPAT-GAN, Coronary-GAN,^4^ and Cycle-GAN. (a) The normal coronary sample. The virtual stained H&E image is very similar to the real H&E image with the three-layer structure resolved. In virtual stained H&E images, the lipid-rich regions appear as white holes. (b) Coronary samples with lipid-rich regions. (c) Coronary samples with calcified regions. The triangle and star represent different texture contrast. The calcified region has a color of dark purple. *The contrasts of OCT images in panels (a)–(c) are enhanced to highlight the texture information for better visualization. The scale bar: 500  μm.

We visually inspect the virtual stained H&E images generated by SCPAT-GAN in Fig. 4. For normal coronary samples, the SCPAT-GAN is capable of generating the three-layer structure; for pathological coronary samples, the SCPAT-GAN is capable of resolving lipid-rich (red arrow) and calcified patterns (yellow star). Compared to real H&E images, virtual stained H&E images generated by SCPAT-GAN show similar patterns for lipid-rich and calcified regions. In contrast, the Coronary-GAN^4^ and Cycle-GAN fail to generate pathological patterns.

**Fig. 5:**
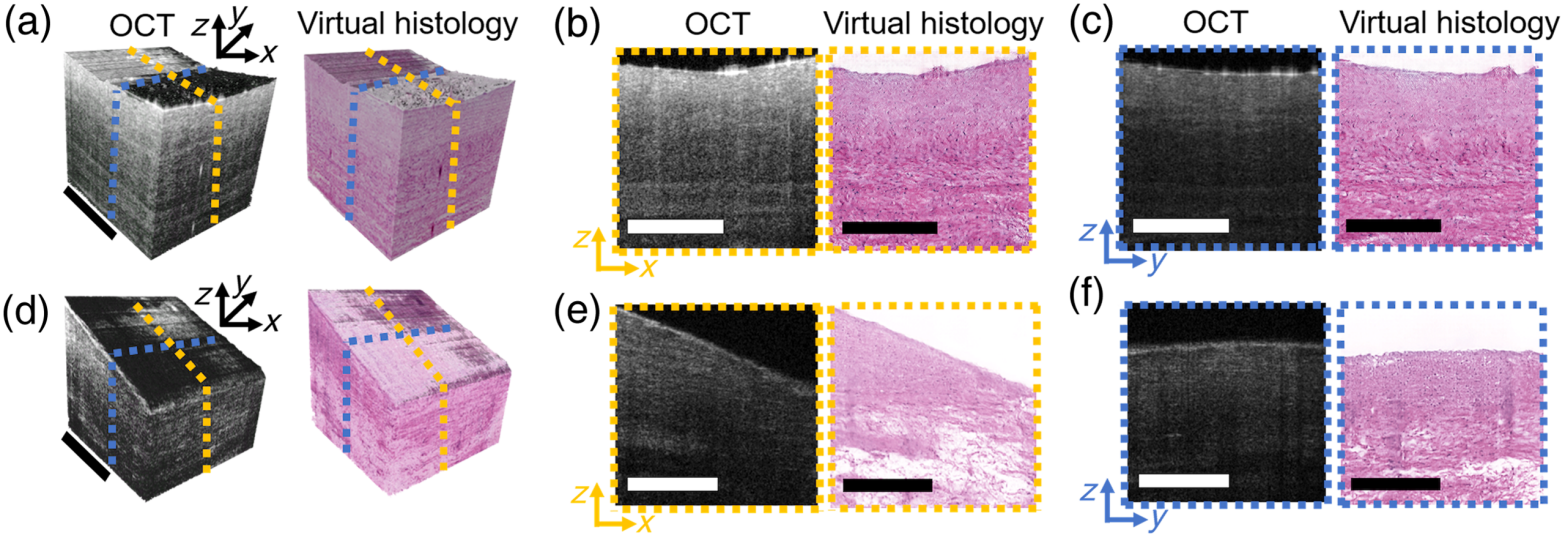
3D virtual stained H&E volumes generated by slice-by-slice from OCT volumes. (a) 3D OCT and virtual stained H&E volumes for a normal human coronary sample. (b) and (c) Example 2D cross-sectional views within (a). (d) 3D OCT and virtual stained H&E volumes for a pathological human coronary sample. (e) and (f): example 2D cross-sectional views within (d). The scale bar: 500  μm.

The proposed SCPAT-GAN allows the generation of 3D virtual H&E volume for both normal and pathological human coronary samples. As shown in Fig. 5, we demonstrate 3D virtual H&E visualization for normal [Fig. 5(a)] and pathological [Fig. 5(d)] coronary samples. The 3D H&E visualization is impossible to acquire from conventional biochemical staining process, which provides an intuitive way of presenting histological information and reduces the randomness of the H&E sanctioning process.^14^

## 4 Discussion

In this paper, we design a convolutional transformer-GAN, namely SCPAT-GAN, for generating virtual stained H&E histology from OCT images. Our SCPAT-GAN algorithm is capable of virtually staining OCT images for human coronary samples. The SCPAT-GAN does not require pixel-wisely matched OCT and H&E datasets. By incorporating structural constraining and pathology awareness functions in the SCPAT-GAN, our method outperforms existing methods, which is confirmed by both objective metrics and the pathologist’s evaluation. Compared to other label-free^15^ or stain-to-stain^8^ works for virtual staining of histology^16^ which focuses on top-view images or other image modalities, our SCPAT-GAN is designed for cross-sectional, depth-resolved OCT images and human coronary samples. Moreover, the proposed SCPAT-GAN is capable of generating 3D virtual stained H&E visualization for coronary samples, which is impossible to acquire using a conventional biochemical staining process.

As the first study to demonstrate the feasibility of virtual stained histology in OCT images from non-paired training, our study does not focus on computational optimization. In the future, we will further reduce the computational overhead of SCPAT-GAN via lightweight neural network^17^ and implement parallel computing for 3D virtual histology. Also, we plan to enable the SCAPT-GAN in intravascular OCT imaging, towards the assistance of percutaneous coronary intervention. Furthermore, we will acquire more data and differentiate pathological patterns to provide fine-grained image-wise labels. Moreover, our current approach still requires image level labels of normal and pathological data and pixel-level layer annotation. We will explore self-supervised approaches to address this issue. Besides, we will explore the other use-cases of the SCPAT-GAN, such as generating multiple types of virtual staining (e.g. Van Gieson staining, Toluidine blue staining, and Alcian blue staining), and virtual staining of other samples (e.g., human skin and eye).

## 5 Conclusion

In this paper, we develop a deep learning model, namely SCPAT-GAN, for generating virtual histology information. Our work is the first to generate virtual H&E images with pathological patterns for coronary samples based on OCT. The proposed framework has great potential to provide real-time histopathological information during an OCT imaging procedure.

## Data Availability

Data underlying the results presented in this paper are not publicly available at this time but may be obtained from the authors upon reasonable request.
